# S100A6 Protein Negatively Regulates CacyBP/SIP-Mediated Inhibition of Gastric Cancer Cell Proliferation and Tumorigenesis

**DOI:** 10.1371/journal.pone.0030185

**Published:** 2012-01-25

**Authors:** Xiaoxuan Ning, Shiren Sun, Kun Zhang, Jie Liang, Yucai Chuai, Yuan Li, Xiaoming Wang

**Affiliations:** 1 Department of Geriatrics, Xijing Hospital, Fourth Military Medical University, Xi'an, Shaanxi, China; 2 Department of Nephrology, Xijing Hospital, Fourth Military Medical University, Xi'an, Shaanxi, China; 3 State Key Laboratory of Cancer Biology and Institute of Digestive Diseases, Xijing Hospital, Fourth Military Medical University, Xi'an, Shaanxi, China; 4 College of Life Science, Shaanxi Normal University, Xi'an, Shaanxi, China; Ospedale Pediatrico Bambino Gesù, Italy

## Abstract

Calcyclin-binding protein (CacyBP/SIP), identified on the basis of its ability to interact with S100 proteins in a calcium-dependent manner, was previously found to inhibit the proliferation and tumorigenesis of gastric cancer cells in our laboratory. Importantly, the effects of S100 proteins on the biological behavior of CacyBP/SIP in gastric cancer remain unclear. Herein, we report the construction of eukaryotic expression vectors for wild-type CacyBP/SIP and a truncated mutant lacking the S100 protein binding domain (CacyBP/SIPΔS100). The expressions of the wild-type and truncated recombinant proteins were demonstrated by transfection of MKN45 gastric cancer cells. Co-immunoprecipitation assays demonstrated interaction between S100A6 and wild-type CacyBP/SIP in MKN45 cells. Removal of the S100 protein binding domain dramatically reduced the affinity of CacyBP/SIP for S100 proteins as indicated by reduced co-immunoprecipitation of S100A6 by CacyBP/SIPΔS100. The MTT assay, FACS assay, clonogenic assay and tumor xenograft experiment were performed to assess the effect of CacyBP/SIP on cell growth and tumorigenesis *in vitro* and *in vivo*. Overexpression of CacyBP/SIP inhibited the proliferation and tumorigenesis of MKN45 gastric cancer cells; the proliferation and tumorigenesis rates were even further reduced by the expression of CacyBP/SIPΔS100. We also showed that S100 proteins negatively regulate CacyBP/SIP-mediated inhibition of gastric cancer cell proliferation, through an effect on β-catenin protein expression and transcriptional activation of Tcf/LEF. Although the underlying mechanism of action requires further investigation, this study provides new insight into the interaction between S100 proteins and CacyBP/SIP, which might enrich our knowledge of S100 proteins and be helpful for our understanding of the development of gastric cancer.

## Introduction

S100 proteins are the largest subgroup within the EF-hand Ca^2+^-binding protein family and are characterized by their cell- and tissue-specific expression patterns. These proteins are known to regulate intracellular processes such as the cell cycle transition and cellular growth, differentiation and motility [1]. A unique feature of these proteins is that individual members are localized in specific cellular compartments from which some are able to relocate upon Ca^2+^ activation [2], [3]. Upon translocation, these proteins may transduce the Ca^2+^ signal in a temporal and spatial manner by interacting with different S100 protein-specific targets. Interestingly, most S100 genes are located in a gene cluster on human chromosome 1q21, a site of frequent chromosomal abnormalities [4]. This association suggests a link between chromosomal abnormalities and the deregulation of S100 gene expression in various tumors. Up to date, many members of S100 family have been identified to be associated with tumor development and metastasis [5], [6]. However, the precise roles and importance of S100 proteins in the development and promotion of cancer are poorly understood. Understanding of the biological function(s) of S100 proteins will depend on the identification of S100 protein targets. Up to now, several possible protein targets, including glyceraldehyde-3-phosphate dehydrogenase, annexin II, annexin VI, annexin XI, caldesmon and CacyBP/SIP, have been shown to interact with S100 proteins *in vitro* in a Ca^2+^-dependent manner [5], [7].

Calcyclin-binding protein (CacyBP/SIP), a 30 kDa protein identified on the basis of its ability to interact with S100A6 in a Ca^2+^-dependent manner in Ehrlich ascites tumour (EAT) cells [8], was previously thought as a multiple-drug resistance (MDR)-related molecule in gastric cancer in our laboratory [9]. Through use of a monoclonal antibody against CacyBP/SIP [10], we have shown that overexpression of CacyBP/SIP inhibits the proliferation and tumorgenesis of renal cancer cells [11]. Further study has suggested that CacyBP/SIP suppresses the growth of gastric cancer [12]. Despite this progress, the effects of S100 proteins on CacyBP/SIP in gastric cancer remain unclear. In addition, CacyBP/SIP was also found to be a component of an ubiquitination complex via binding Siah1 and Skp1 [13]. And this ligase was described to regulate the degradation of non-phosphorylated β-catenin [13].

CacyBP/SIP could bind S100, Siah1 and Skp1 by different regions. The N-terminal region (aa 1–80) of CacyBP/SIP has been shown to have affinity for Siah1. The C-terminal region of CacyBP/SIP (aa178–229) is known to form a α-helix; this domain may interact with S100 proteins, including S100A6 [14]. Skp1 binding domain does not overlap with the S100 binding region and Skp1 binds to the middle part (aa78–155) of CacyBP/SIP [15], [16]. To observe the effects of S100 proteins on the biological behavior of CacyBP/SIP in gastric cancer, eukaryotic expression vectors for wild-type CacyBP/SIP and its truncated mutant lacking the S100 protein binding domain (CacyBP/SIPΔS100) were successfully constructed and effectively expressed in MKN45 cells. An MTT assay, FACS assay, clonogenic assay and tumor xenograft experiment were performed to evaluate the effects of CacyBP/SIP on cell growth and tumorigenesis both *in vitro* and *in vivo*. The results of these studies may contribute to the elucidation of the effects of S100 proteins on the biological behavior of CacyBP/SIP in gastric cancer cells.

## Materials and Methods

### Cell lines and animals

The gastric cancer cell line MKN45, which was found to be the lowest expression of CacyBP/SIP in our previous study [12], was preserved in our laboratory. The cells were cultivated in RPMI 1640 medium (GIBCO, Grand Island, NY, USA) supplemented with 10% heat-inactivated fetal calf serum, penicillin (100 U/ml), and streptomycin (100 µg/ml) in a CO_2_ incubator (Forma Scientic, Marjetta, OH, USA). BALB/c nude mice at 4–6 weeks of age were provided by the Shanghai Cancer Institute for the *in vivo* tumorigenesis study. This study was performed in strict accordance with the recommendations in the Guide for the Care and Use of Laboratory Animals of the National Institutes of Health. The protocol was approved by the Committee on the Ethics of Animal Experiments of the Fourth Military Medical University (Permit Number: 11016). All surgery was performed under sodium pentobarbital anesthesia, and every effort was made to minimize suffering.

### Immunofluorescence staining

The cells were plated onto cleaned coverslips and fixed with 95% alcohol for 20 min at room temperature. The cells on the coverslips were washed with PBS and permeablized for 10 min with 0.5% Triton X-100 in PBS. The cells were incubated with anti-S100A6 antibody (diluted 1∶2000) after blocking with 3% bovine serum albumin for 1 h. The cells were then incubated with FITC-conjugated anti-mouse IgG (Santa Cruz Biotech., Santa Cruz, USA) and mounted on glass slides with a mixture of glycerol and polyvinyl alcohol containing DABCO (1,4- diazobicylo-[2,2,2,]-octane). Immunofluorescence was analyzed with a MRC-1024 Laser Scanning Confocal Imaging System (Bio-Rad).

### Plasmid construction and cell transfection

Oligonucleotide primers containing the *Eco*R I or *Eco*R V restriction site were synthesized, respectively, for amplification of the CDS sequence of CacyBP/SIP or a C-terminal deletion (aa178–229) mutant of CacyBP/SIP (Accession AF_314752). The two primers were: 5′ gggaattcgaatatggcttcagaagagcta 3′ (sense) and 5′ gcgatatctcaaaattccgtgtctcctttg 3′ (anti-sense); 5′ gggaattcgaatatggcttcagaagagcta 3′ (sense) and 5′ ccgatatcacacaatataagaactgta 3′ (anti-sense), respectively. The PCR conditions were: 5 min at 94° for initial denaturation, followed by 35 cycles of 45 sec at 94°, 45 sec at 60°, and 60 sec at 72°, with a final extension of 10 min at 72°. The lengths of the PCR products were 687 bp for CDS, and 534 bp for C-terminal deletion. Each PCR product was excised with *EcoR* I and *EcoR* V restriction digestion and cloned into likewise digested pFLAG-CMV. The new vectors were named pFLAG-CacyBP (wild-type) and pFLAG-ΔS100 (CacyBP/SIPΔS100). The insert sequences were confirmed by DNA sequencing.

Cell transfection was performed with Lipofectamine 2000 (Invitrogen, Carlsbad, CA) as described by the manufacturer. Briefly, cells were plated and grown to 70–90% confluence without antibiotics. They were then transfected with 1 µg pFLAG-CacyBP or pFLAG-ΔS100. At 24 h after transfection, G418 (350 mg/ml) was added to the culture media for the selection of transfected cells. Mixed clones were expanded for an additional 6 weeks. Cells transfected with empty vector, pFLAG-CMV, were used as a negative control. These stable cell lines were named MKN45-CacyBP, MKN45-ΔS100 and MKN45-pFLAG for the wild-type, truncated and negative control transfectants, respectively.

### Co-immunoprecipitation

The MKN45 transfectants were washed in PBS, harvested and lysed on ice in a buffer containing 20 mM Tris-HCl (pH 7.5), 8 mM MgCl_2_, 150 mM NaCl, 0.2 mM EGTA, and 1% Nonidet P-40. The extracts were centrifuged at 12,000 rpm for 25 min at 37° in a microcentrifuge. The supernatants were used for an immunoprecipitation assay after the addition of protease inhibitors (10 mg/L leupeptin, 5 mg/L aprotinin, 20 mg/L soybean trypsin inhibitor, 1 mM phenylmethylsulfonyl fluoride) and quantified by the Bradford method. The supernatants were incubated with 30 µl protein A/G-Sepharose and 1 µg unrelated antibody for 1–3 h at 37° (pre-clearance). The unbound fractions were then incubated with serum containing antibodies against CacyBP/SIP for 1.5 h at 4°. The mixture was then incubated with additional protein A/G-Sepharose overnight at 4°. The resin was then washed three times in a buffer containing 20 mM Tris- HCl (pH 7.5) and 150 mM NaCl, twice in a buffer containing 20 mM Tris-HCl (pH 7.5) and 500 mM NaCl, and finally in 20 mM Tris-HCl at pH 7.5. All of the buffers that were used were supplemented with protease inhibitors. The resin and bound proteins were solubilized in an SDS sample buffer, boiled for 5 min at 98°, and applied to an SDS polyacrylamide gel. The expression of S100A6 was analyzed by western blotting with antibody against S100A6.

### Western blot

The cells were collected and lysed on ice in a buffer containing [50 mmol/L Tris-Cl (pH 7.5), 150 mmol/L NaCl, 0.2 mmol/L EDTA, 1 mmol/L PMSF and 1% NP 40], and then quantified by the Bradford method. A measure of 100 µg of lysates was electrophoresed in 10% SDS-PAGE and blotted on to a nitrocellulose membrane. The membranes were blocked with 10% fat-free milk at room temperature for 2 h and incubated with anti-CacyBP (1∶1500) and anti-β-actin antibody (Sigma, 1∶5000) at 4° overnight. After three washes for 15 min each in TBS supplemented with 0.1% Tween 20 (TBST), the membrane was incubated with peroxidase-conjugated goat anti-mouse/rabbit IgG antibody (Amersham-Pharmacia Biotech, Beijing, China) for 2 h at room temperature. Enhanced chemiluminescence (Amersham, Freiburg, Germany) was used for detection.

### Methyl thiazolyl tetrazolium (MTT) assay

Cells were seeded onto a 96-well plate at 2.5×10^3^ cells/well in RPMI 1640 media containing 10% heat-inactivated fetal calf serum. Each sample had three replicates. The medium was replaced at 2-day intervals, for 6 days. The cells were incubated with 50 µl of 0.2% MTT for 4 h at 37° in a 5% CO_2_ atmosphere. Following MTT incubation, a 150 µl aliquot of 100% DMSO was added to each culture to dissolve the crystals. Viable cells were counted every day by reading the absorbance at 490 nm using a 96-plate reader, BP800 (Dynex Technologies, Chantilly, VA).

### Cell cycle analysis

Subconfluent cells were washed with ice-cold PBS, suspended in 0.5 ml ethanol and then kept at 4°C for 30 min. The suspension was filtered through a 50-µm nylon mesh, and the DNA content of stained nuclei was analyzed with a flow cytometer (EPICS XL, Coulter, Miami, FL). The cell cycle profile was analyzed using Multicycle-DNA Cell Cycle Analyzed Software. The proliferative indices (PI) were calculated as follows: PI = (G2+S)/(G1+S+G2).

### Clonogenic assay

To measure the proliferation rate of a single cell *in vitro*, plate clonogenic and soft agar clonogenic assays were performed [17]. For the plate clonogenic assay, 1×10^3^ cells were seeded into a 9-cm dish and cultured in RPMI 1640 medium for two weeks to allow for colony formation. The colonies were fixed in 70% ethanol, stained with Giemsa solution and counted. For the soft agar clonogenic assay, the cells were detached and plated in 0.3% agarose with a 0.5% agarose underlay (1×10^3^/well in a 24-well plate). The number of foci >100 µm was counted after 20 days.

### Tumor growth in nude mice

Logarithmically growing cells (1×10^7^ cells) were trypsinized, resuspended in 0.1 ml D'Hanks solution, and injected into the neck hypodermia of each athymic nude mouse. The mice were then monitored for tumor volume, overall health, and total body weight. The size of the subcutaneous tumor mass was measured by caliper for five weeks. Tumor volume was calculated according to the formula: 0.5×length×width^2^. Each experimental group consisted of five mice.

### Reporter gene assay

To assay the effect of CacyBP/SIP and CacyBP/SIPΔS100 on the transcriptional activity of Tcf/LEF, the reporter gene assay was performed as described [18]. Briefly, cells in a 24-well plate (50,000 cells per well) were co-transfected with or without pFLAG-CMV, pFLAG-CacyBP, pFLAG-ΔS100 and the reporter plasmid, pTOPFLASH, containing wild-type Tcf/LEF binding sites using Lipofectamine 2000; pRL-TK *Renilla* luciferase reporter served as a control for transfection efficiency. The luciferase activity was measured and quantitated in a luminometer using the Dual-Luciferase Reporter Assay System (Promega, Southampton, U.K.). Experiments were performed in triplicate and repeated twice. Results are expressed as the mean of the ratio between the firefly luciferase activity and the renilla luciferase activity.

### Statistical analysis

All data were analyzed using the SPSS statistical software package (SPSS, Inc., Chicago, Illinois), and *P*<0.05 was considered statistically significant. The significance of the difference in frequency of CacyBP/SIP-positive staining between adjacent tumor samples and tumors were analyzed by the chi-square test. Student's *t* test and one-way analysis of variance followed by Dunnett's multiple comparison tests were employed for analysis of other data.

## Results

### Endogenous expression of S100A6 in gastric cancer cell line MKN45

**Figure 1:**
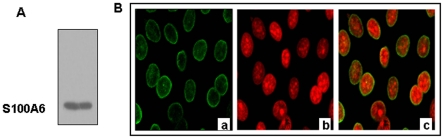
Endogenous expression of S100A6 in the MKN45 gastric cancer cell line. (A) Endogenous expression of S100A6 in MKN45 gastric cancer cells was detected by western blot. (B) Immunofluorescent staining of S100A6 in MKN45 cells. (a) Immunofluorescent staining of S100A6 with anti-S100A6 antibody (in green); (b) Detection of nuclei with propidium iodide (in red); (c) Merged picture.

S100A6 was selected as a model to observe the effect of S100 proteins on the biological function of CacyBP/SIP. The endogenous expression of S100A6 in gastric cancer cell line MKN45 was first detected by western blot and immunofluorescence staining. As shown in Fig. 1A, S100A6 was expressed in MKN45 cells. Immunofluorescence staining indicates that S100A6 was mainly distributed in the nuclear membrane with very little found in the nuclear plasma of the MKN45 cells (Fig. 1B).

### Interaction of S100A6 and CacyBP/SIP in gastric cancer cells MKN45

**Figure 2:**
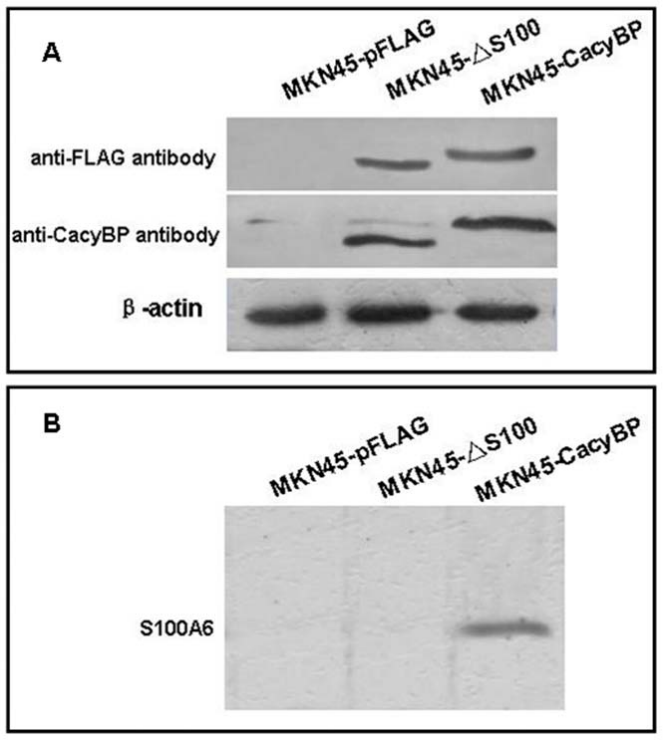
Interaction of S100A6 and CacyBP/SIP in the MKN45 gastric cancer cell line. (A) Western blot analysis of the transfectants CacyBP/SIP and CacyBP/SIPΔS100 transfectants with anti-FLAG and anti-CacyBP antibodies. β-actin was used as a loading control. (B) Co-immunoprecipitation assay to detect an interaction between S100A6 and CacyBP/SIP in MKN45 cells. After incubation with anti-CabyBP antibody, the binding sites were detected by anti-S100A6 antibody.

In a previous report, CacyBP/SIP, which was expressed at a relatively low level in MKN45 cells, was widely expressed in several different types of gastric cancer cell lines [12]. In order to evaluate the interaction between S100A6 and CacyBP/SIP, wild-type and mutant (aa178–229) CacyBP/SIP cDNAs were cloned into the pFLAG-CMV vector and stably transfected into MKN45 cells. We assessed the expression of CacyBP/SIP in these stable cell lines by western blot. As shown in Fig. 2A, FLAG-tagged CacyBP/SIP was detected in the wild-type and mutant CacyBP/SIP transfectants with anti-Flag antibody, while no signal was found in cells transfected with empty vector.

The interaction between S100A6 and CacyBP/SIP in transfectant MKN45 cells was then evaluated by co-immunoprecipitation. After incubation with anti-CacyBP antibody, immunoprecipitate were detected by S100A6 antibody. As shown in Fig. 2B, an interaction between S100A6 and wild-type CacyBP/SIP was observed in MKN45-CacyBP cell lysates; however, mutant CacyBP/SIP that was truncated from the aa178–229 region, produced little signal in the co-immunoprecipitation assay. Therefore, the truncated mutant CacyBP/SIP was named CacyBP/SIPΔS100 and provides a useful tool for investigation of the effects of S100A6 on the biological behavior of CacyBP/SIP in gastric cancer cells.

### Effects of S100A6 on cell proliferation induced by CacyBP/SIP in the gastric cancer cell line MKN45 *in vitro*

**Figure 3:**
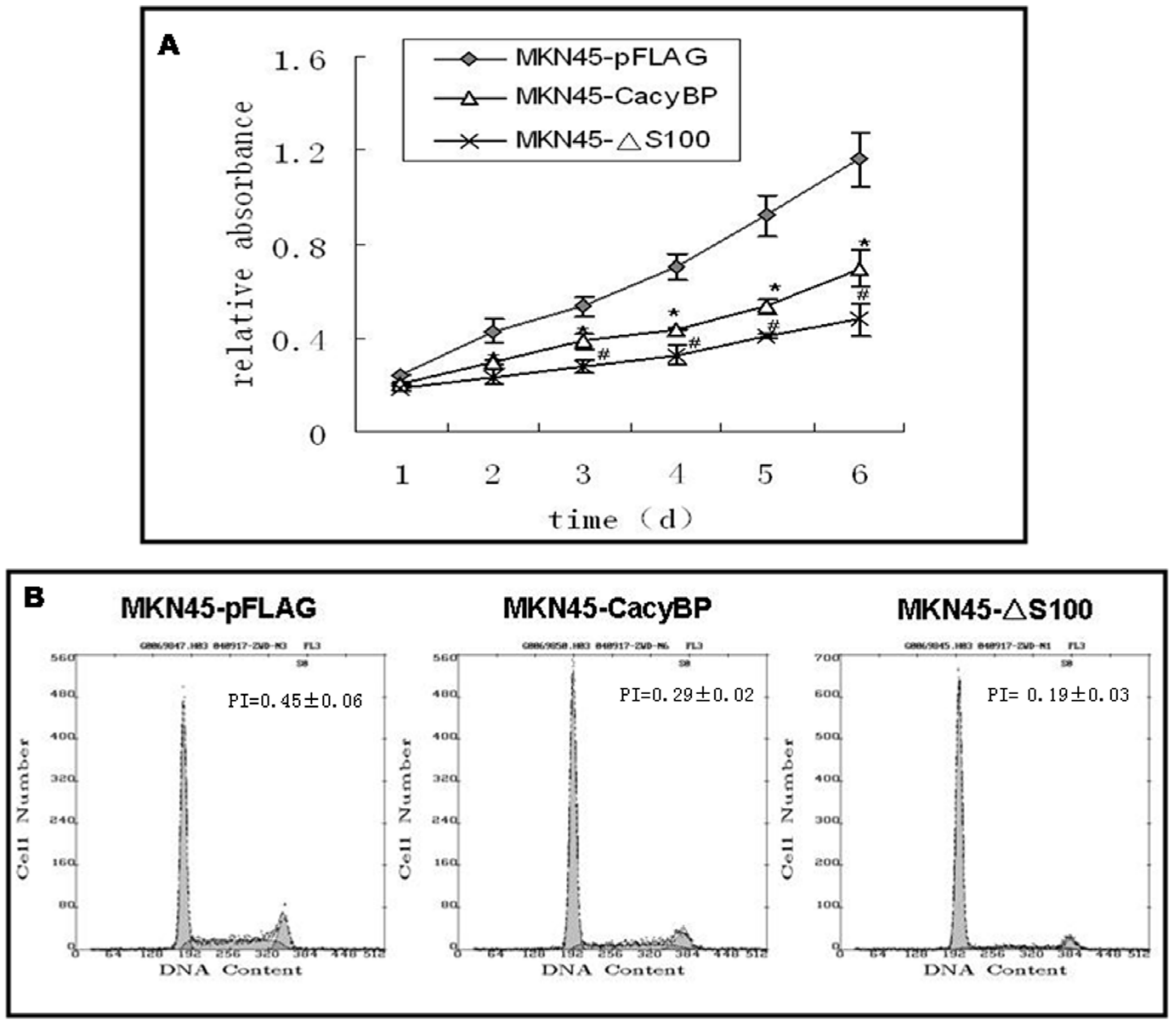
Effects of CacyBP/SIP on cell proliferation *in vitro*. (A) Detection of the cell growth rate *in vitro*. Cell number was evaluated by absorbance at 490 nm using the MTT assay at the indicated times. The value shown is the mean of three determinations. (B) Cell cycle distribution and proliferative indices (PI) of the transfected cells. The cells were detergent extracted, stained with propidium iodide, and analyzed by flow cytometry. PI = (G2+S)/(G1+S+G2).

MTT and FACS assays were performed to examine the effects of S100A6 on cell proliferation induced by CacyBP/SIP in MKN45 cells *in vitro*. Compared with the empty vector transfectants, MKN45-pFLAG, both wild-type and CacyBP/SIPΔS100 transfectants showed significantly decreased rates of cell proliferation by the MTT assay (*P*<0.05) (Fig. 3A). However, CacyBP/SIPΔS100 transfectants exhibited a significantly more decreased growth rate compared with wild-type CacyBP/SIP transfectants (*P*<0.05).

The results of the cell cycle analysis by FACS similarly indicate that overexpression of either wild or mutant CacyBP/SIP reduces MKN45 proliferation. However, the average proliferative indexes (PI) of MKN45-ΔS100 cells (0.19±0.03) was lower than that of MKN45-CacyBP cells (0.29±0.02) (*P*<0.05) (Fig. 3B). Taken together, these data strongly indicate that wild-type CacyBP/SIP inhibits the proliferation of MKN45 cells, while CacyBP/SIPΔS100 intensifies this inhibition of proliferation.

### Effects of S100A6 on tumorigenesis induced by CacyBP/SIP in the MKN45 gastric cancer cell line *in vitro* and *in vivo*

**Figure 4:**
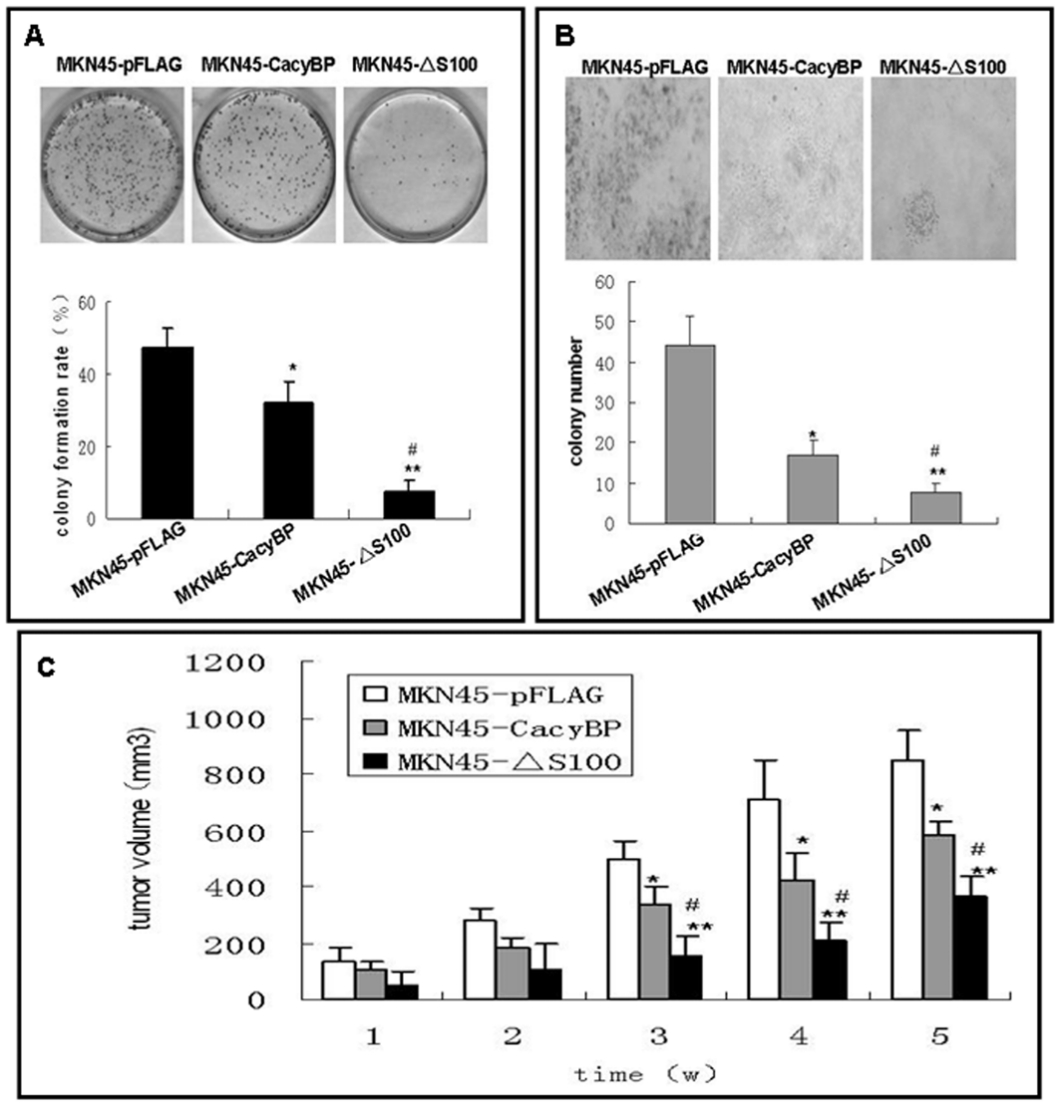
Effects of CacyBP/SIP transfectants on tumorigenesis. (A) Detection of the clonogenic formation in medium. (B) Detection of the clonogenic formation in soft agar. Cells were placed in media containing soft agar or on a plate and incubated for 20 days. The number of foci >100 µm was counted. Vertical bars represent the mean ± SD from at least three separate experiments, each conducted in triplicate. (C) *In vivo* tumorigenicity was evaluated by a nude mice assay. Mice were injected subcutaneously with 1×10^7^ transfected cells. The volume of each tumor was calculated according to the formula 0.5×length×width^2^. Two independent experiments were performed and each experimental group consisted of five mice. ^*^ *P*<0.05 *vs.* MKN45-pFLAG, ^**^ *P*<0.01 *vs.* MKN45-pFLAG, ^#^ *P*<0.05 *vs.* MKN45-CacyBP.

Anchorage-independent growth is an important characteristic of *in vitro* tumor growth. Therefore, we examined whether upregulation of CacyBP/SIP expression inhibited MKN45 cell growth in media and soft agar. As shown in Fig. 4A and B, CacyBP/SIP expression resulted in a marked reduction of MKN45 cell growth in a colony formation assay, with an average reduction of 32.1% in medium and 62.0% in soft agar (*P*<0.05). However, transfection of CacyBP/SIPΔS100 resulted in an even greater decrease in growth with an average decrease in rates of 84.2% in medium and 82.8% in soft agar (*P*<0.01).

To confirm this effect *in vivo*, we subcutaneously injected MKN45-CacyBP, MKN45-ΔS100 and MKN45-pFLAG cells in to the neck hypodermia of nude mice. After three weeks, the average tumor volume in each group was significantly different from that of the others. At the 5^th^ week, the average tumor volume in the MKN45-pFLAG group was 776.9±90.9 mm^3^, which is significantly more than that of the MKN45-CacyBP group (578.9±51.2 mm^3^) (*P*<0.05), and even more than that of the MKN45-ΔS100 group (364.9±71.9 mm^3^) (*P*<0.05) (Fig. 4C).

### Effects of S100A6 on CayBP/SIP-mediated β –catenin degradation

**Figure 5:**
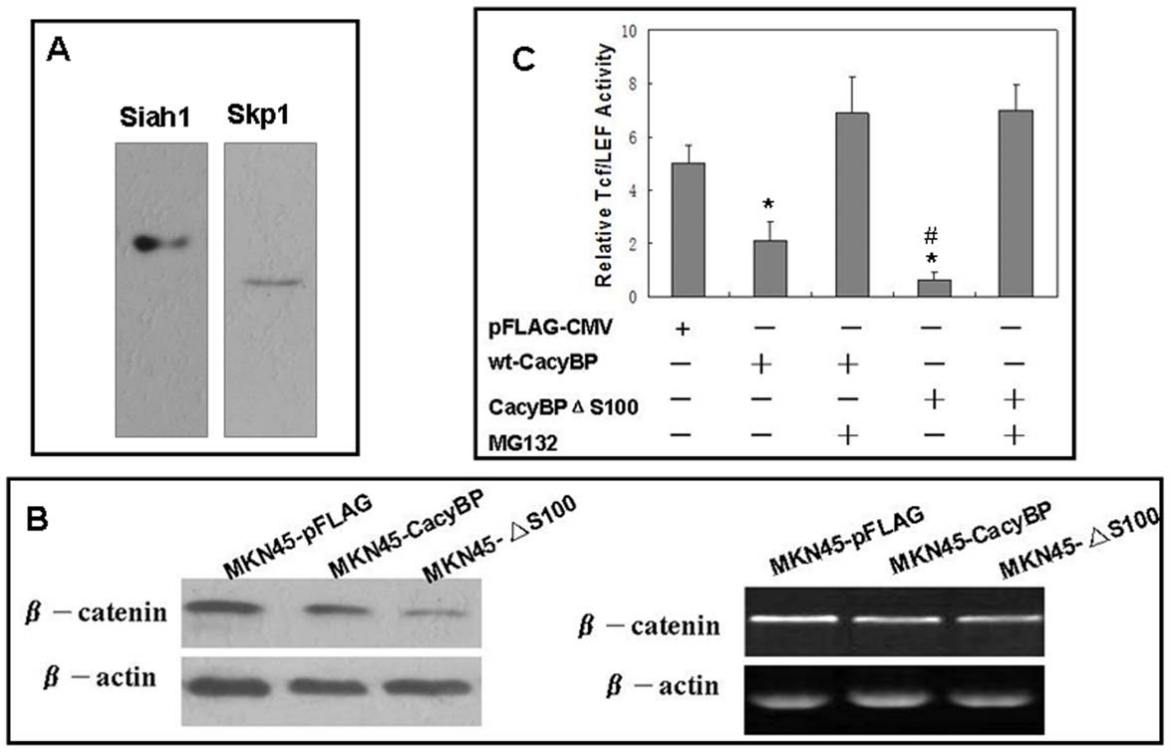
Effects of S100A6 on CayBP/SIP-mediated β –catenin degradation. (A) Co-immunoprecipitation assay showed that truncated mutant CacyBP/SIPΔS100 bind both Skp1 and Siah1, suggesting S100A6 did not affect the formation of Siah1-CacyBP/SIP-Skp1 unbiquitin ligase complex. (B) Western blot and semi-quantitative RT-PCR were used for analysis of β-catenin protein and mRNA in transfected cells, respectively. β–catenin protein in MKN45-ΔS100 cells is significantly decreased, compared to MKN45-CacyBP cells, while with no change occurred in the mRNA. (C) The luciferase reporter gene assays were used to evaluate effect of CacyBP/SIPΔS100 on Tcf/LEF reporter activity. Transfection of wild-type CacyBP/SIP decrease the Tcf/LEF activity, compared to control plasmid. And expression of CacyBP/SIPΔS100 resulted in lower Tcf/LEF activity than wild-type CacyBP/SIP. MG132 (inhibitor of proteasome) could eliminate the effect of both wild-type CacyBP/SIP and CacyBP/SIPΔS100 on Tcf/LEF activity. *P<0.01, compared with the control; ^#^ *P*<0.05, CacyBP/SIPΔS100 *vs.* wild-type CacyBP/SIP. These experiments were repeated in triplicate.

As a component of ubiquitin ligase complex, CacyBP/SIP is involved in the degradation of unphosphorylated β –catenin via binding Skp1 and Siah1. So expression of β –catenin was detected to indirectly evaluate the influence of S100 on Siah1-CacyBP/SIP-Skp1 unbiquitin ligase. Firstly, co-immunoprecipitation assay showed that truncated mutant CacyBP/SIP bind both Skp1 and Siah1 (Fig. 5A), suggesting S100A6 did not affect the formation of Siah1-CacyBP/SIP-Skp1 unbiquitin ligase complex. Secondly, β– catenin protein in MKN45-ΔS100 cells is significantly decreased, compared to MKN45-CacyBP cells, while with no change occurred in the mRNA (Fig. 5B). In addition, because β-catenin is required as a cofactor for the activation of the transcription factor, Tcf/LEF, we determined the effects of CayBP/SIPΔS100 on Tcf/LEF activity using transient transfection reporter gene assays. Expression of CacyBP/SIPΔS100 resulted in lower Tcf/LEF activity than wild-type CacyBP/SIP (Fig. 5C). MG132, inhibitor of proteasome, could eliminate the effect of both wild-type CacyBP/SIP and CacyBP/SIPΔS100 on Tcf/LEF activity. Taken together, we inferred that S100A6 might affect CayBP/SIP mediated β –catenin degradation via interacting with CayBP/SIP.

## Discussion

S100 family proteins have gained interest because of their differential expression in tumor tissues and their involvement in cancer progression and metastasis [19]–[22]. S100 proteins interact with some target proteins in calcium-dependent or calcium-independent manner and further regulate function of these targets [5], [23]. CacyBP/SIP is a new target protein that can interact with S100 proteins, including S100A6, S100A1, S100B, and S100P, but not to S100A4, calbindin D, parvalbumin or calmodulin [8], [16]. Previous works in our lab showed that overexpression of CacyBP/SIP could inhibit the proliferation and tumorigenesis of gastric cancer and renal cell carcinoma [11], [12]. Despite this progress, the effects of S100 proteins on CacyBP/SIP remain to be investigated.

To elucidate the regulation of S100 proteins on CacyBP/SIP function, we constructed the truncated CacyBP/SIP mutant without S100 protein binding domain, so that mutant protein could not interact S100 proteins and retains ability to interact with Siah1 and Skp1. In this study, eukaryotic expression vectors for wild-type CacyBP/SIP and its truncated mutant were effectively expressed in MKN45 cells. Co-immunoprecipitation demonstrated an interaction between S100A6 and wild-type CacyBP/SIP in MKN45 cells. However, CacyBP/SIPΔS100 showed little signal by the co-immunoprecipitation assay, indicating a weak or no interaction with S100A6. MTT assay, FACS assay, clonogenic assay and tumor xenograft experiment in nude mice were performed to test the effect of wild and mutant CacyBP/SIP on cell growth and tumorigenesis both *in vitro* and *in vitro*. These experiments showed that overexpression of CacyBP/SIP could inhibit the proliferation and tumorigenesis of MKN45 gastric cancer cells. Importantly, cells transfected with CacyBP/SIPΔS100 exhibited more intense effects relative to wild-type transfectants, suggesting that S100 proteins might negatively regulate the inhibition of cell proliferation induced by CacyBP/SIP in gastric cancer cells.

The mechanism employed by S100A6 to modulate CacyBP/SIP function might lie in protein interactions and functions. CacyBP/SIP is identified to be involved in ubiquitination and degradation of β-catenin [24]–[28]. β- catenin was found to be overexpressed or activated in proliferating cells and in many tumors [29]–[31]. Fukushima et al. [32] found that CacyBP/SIP knockout cells show impaired (-catenin degradation and a defective G1 cell cycle checkpoint, indicating that the β-catenin degradation pathway mediated by CacyBP/SIP defines an essential checkpoint for thymocyte development and cell cycle progression. Our previous studies also showed that CacyBP/SIP inhibits growth and proliferation of gastric cancer cells partly via degradation of β –catenin. Whether S100 regulates CacyBP/SIP-mediated ubiquitin ligase retains unknown. Accumulating evidences showed that several members of the S100 protein family could regulate the activation of their target proteins [33], [34]. A recently discovered yeast homolog of CacyBP/SIP, Sgt1, not only interacts with S100 proteins in calcium regulated-manner, but also associates with Skp1 to form Skp1/cullin1/F- box ubiquitin ligase complex [35]. It has been found that S100A6 could inhibit the phosphorylation of Sgt1 by casein kinase II to affect the activity [36]. In our present experiment, we also identified that CacyBP/SIPΔS100 retain ability to associate with Skp1 and Siah1, suggesting S100 molecule might not affect formation of Siah1/Skp1/CacyBP ubiquitin ligase complex. Nevertheless, β–catenin protein without a change of the mRNA level in CacyBP/SIPΔS100 transfected cells is markedly lower than wild-type transfectant cells. The transcriptional activity of Tcf/LEF, a target gene of β–catenin, was decreased in conjunction with mutant CacyBP/SIP without S100 binding, compared to wild CacyBP/SIP. Accoding to these results, we postulate that S100 protein might negatively regulate the activity of Siah1-CacyBP/SIP-Skp1 ubiquitin ligase by interaction with CacyBP/SIP. So blocking the combination of S100 and CacyBP/SIP could promote β –catenin degradation, result in inhibiting the proliferation of cancer cells. This speculation can be partially supported by Lee et al that in cells with diminished level of S100A6 the amount of β –catenin is reduced [26]. The results are in agreement with data regarding the up-regulation of S100 proteins and deficient degradation of β –catenin in tumor and proliferating cells [37]. In addition, CacyBP/SIP was shown to interact with tubulin and ERK1/2 and play important role in rearrangements of the cytoskeleton, cell differentiation or degeneration [38]–[42].

### Conclusions

S100A6 protein negatively regulates CacyBP/SIP-mediated inhibition of gastric cancer cell proliferation, partly through effect on β-catenin degradation and transcriptional activation of Tcf/LEF. However, the underlying mechanisms for regulation of S100A6 on protein ubiquitination and other functions via CacyBP/SIP-dependent pathway require further investigation.
